# Creatine phosphokinase, a new marker in diagnosis of tubal ectopic pregnancy; A Systematic Review 

**Published:** 2020-01-11

**Authors:** Maryam Ghorbani, Afsaneh Keramat, Farideh Mohsenzadeh Ledari

**Affiliations:** 1 Student Research Committee, School of Nursing and Midwifery, Shahroud University of Medical Sciences, Shahroud, Iran.; 2 Reproductive Studies and Women’s Health Research Center, Shahroud University of Medical Sciences, Shahroud, Iran.; 3 Infertility and Health Reproductive Research Center, Health Research Institute, Babol University of Medical Sciences, Babol, Iran.

**Keywords:** Pregnancy, ectopic, early diagnosis, creatine kinase, biomarkers

## Abstract

**Introduction:**

Creatine phosphokinase (CPK) is an intracellular enzyme found in higher levels in the brain, myocardium, soft muscle and skeletal muscle, as well as the fallopian tube. This review was conducted to evaluate the role of serum CPK in early diagnosis of tubal ectopic pregnancy (EP).

**Methods:**

We performed an electronic literature search in Web of Science, Scopus, Embase and Medline databases and manual search in Google scholar and evaluated papers from the beginning of 1990 to September 2018. The inclusion criteria consisted of cohort, case-control and diagnostic value studies in English or Persian. Two independent researchers used the inclusion and exclusion criteria. In cases where there was doubt about the eligibility of studies, this problem was resolved by consulting a third researcher. After a thorough search, finally, we found 27 papers. However, four of these articles did not have the inclusion criteria and we excluded them from the study. As a result, 24 studies were evaluated.

**Results:**

Most studies have approved the use of CPK measurements in EP diagnosis. The main variable measured in most studies was the mean total CPK level. However, there is limited knowledge about the efficacy of measuring CPK levels in EP diagnosis; this review of studies shows positive results regarding use of CPK in EP diagnosis.

**Conclusions:**

The results highlighted the potential benefits of CPK as a marker for early diagnosis of EP.

## Introduction:

Ectopic pregnancy (EP) occurs when a blastocyst abnormally implants outside the endometrium of the uterus (1). It implants in the fallopian tube in more than 95% of cases (2). This disorder is a major health problem worldwide (3). The prevalence of EP has doubled since 1960 and accounts for about 2% of the pregnancies in the first trimester (1). In recent years, its incidence has increased due to the increase in incidence of pelvic inflammatory diseases, use of fertility drugs, and pelvic surgery (2). Although maternal mortality due to ectopic pregnancy has decreased recently, it is still one of the leading causes of death in the first trimester of pregnancy; hence, early management of ectopic pregnancy is very important (3). In developing countries, such as Iran, 10% of women diagnosed with ectopic pregnancy do not survive because they refer to the hospital very late (1).

Clinical manifestations in ectopic pregnancy can be similar to other conditions. This reveals the need to search for some new diagnostic tools. Transvaginal ultrasound and serial measurement of serum beta-hCG levels are the most common diagnostic methods for EP (4). Despite the use of transvaginal ultrasound and measurement of beta-hCG levels, about 40% to 50% of the initial cases of the disease are not diagnosed. Transvaginal ultrasonography can help if there is an intrauterine pregnancy or an adnexal mass, and measurement of serum beta-hCG levels can detect a normal intrauterine pregnancy from a nonviable pregnancy, but it cannot differentiate an intrauterine pregnancy that has stopped growing from an ectopic pregnancy (5). Creatine phosphokinase (CPK) was proposed as a new EP diagnostic criterion. Lavie et al. were the first to report a sensitivity and specificity for overall CPK levels in detecting EP (2). CPK is an intracellular enzyme found in higher levels in the brain, myocardium, soft muscle and skeletal muscle, as well as the fallopian tube (4). CPK has three definite Isozymes, including CPK-MM, MB, and BB. Due to the lack of a submucosal layer in the fallopian tube, the zygote implants are placed adjacent to the muscle layer in tubal ectopic pregnancy, and this invasion leads to an increase in the level of CPK as a soft muscle damage marker (2).

Due to the need for and importance of early detection and timely treatment of EP and the ambiguousness of ultrasound in most patients, this review is conducted to evaluate the role of serum CPK in early diagnosis of tubal ectopic pregnancy. 

## Methods:

In this systematic review, researchers performed an electronic search using the keywords of Tubal pregnancy, Ectopic pregnancy, EP, Creatinine phosphokinase, and CPK in Medline (via PubMed), Embase, Web of Science, and Scopus databases. Manual search was also performed in Google scholar to find relevant papers. We included articles published from the beginning of 1990 to September 2018. Search terms were selected based on common keywords in literature. The keywords were combined using (AND) and (OR) operators. We used the following search strategy for finding articles with related titles and abstracts in PubMed: ((("Creatine Kinase"[Mesh] OR "Creatine Kinase, MB Form"[Mesh]) AND "Pregnancy, Tubal"[Mesh]) AND "Pregnancy, Ectopic"[Mesh]) AND ("1980/01/01"[PDAT]: "2018/12/31"[PDAT]). 

**Search strategy**

The inclusion criteria consisted of cohort, case-control and diagnostic accuracy studies in English or Persian. Exclusion criteria consisted of articles in languages other than English and Persian, and lack of reporting the data necessary for the study.

**Quality control of the study**

Two independent researchers searched the articles in accordance with inclusion and exclusion criteria. In cases where there was disagreement about the eligibility of studies, we solved the problem by consulting a third researcher. As a result, this review used 24 studies to evaluate the value of CPK in diagnosis of EP. 

This systematic review was reported according to PRISMA checklist criteria. The methodological elements of the study, including data extraction, proper sampling description, study design, participants’ characteristics, and inclusion and exclusion criteria were evaluated to ensure the quality of the selected papers. Two researchers searched and extracted data independently. QUADAS-2 checklist was used for controlling the selected studies. This tool comprises four domains: patient selection, index test, reference standard, and flow and timing. Each domain is assessed in terms of risk of bias, and the first three domains are also assessed in terms of concerns regarding applicability. Signaling questions are included to help judge risk of bias.

## Results:

**Figure 1:**
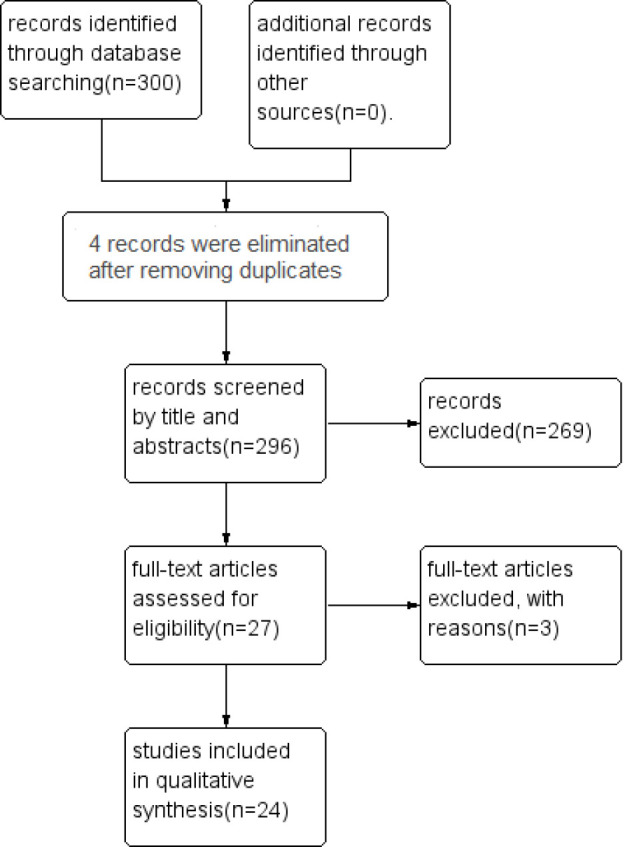
PRISMA flow diagram of the study selection process.

After a thorough search, we found 27 papers on CPK as a marker in diagnosis of tubal EP. However, three of these articles did not meet the inclusion criteria and we excluded them from the study for reasons including failure to report the complete information for the study, type of study, and the language of the study. As a result, 24 studies (2, 4, 6-27) were used to evaluate the value of CPK in diagnosis of EP (Figure 1). 

**Quality assessment of studies and risk of bias**

**Figure 2:**
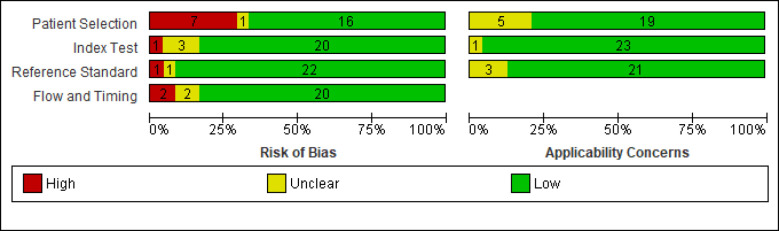
Risk of bias and applicability graph.

The risk of bias in index test, flow and timing and reference standard were low in most studies (83.3% to 91.66% of articles), but in patient selection, 29.11% of articles were high risk, 4.16% of them had unclear risk, and 66.66% had low risk. There were no high applicability concerns regarding patient selection, index test and reference standard among articles. Figure 2 and 3 show the result of quality assessment of studies. 

Table 1 shows the general characteristics and data of each of the articles that were included in the study. 

Total sample size in these 24 articles was 2180 cases in different groups of patients. In all of them the control groups were normal/ intrauterine pregnancy or non-Ep groups and in most of them there was also a third control group, which was abortion cases (2, 4, 9-17, 19, 21, 22, 26, 27). In a study by Chandra & Jain in 1995, two other control groups were also included; acute appendicitis and pelvic inflammatory disease. The result of this study showed significant difference in CPK level of EP group and these groups of patients (11). Most studies have approved the use of serum CPK measurement in EP diagnosis and showed significant difference between ectopic pregnancies and intrauterine pregnancies (2, 4, 6, 7, 9-11, 16, 18, 19, 22-27) but six studies did not show any significant difference (8, 12-15, 17) . The main variable measured in most studies was the mean total CPK level, which had a wide range in these articles; ranging from 33.4 to 185.6 IU/L (Table 1). Reference tests for confirming ectopic pregnancy in almost all of studies were B-hCG blood sampling and/or ultrasonography. 

Mean reported level of CPK was 95.02±51.09 IU/L for ectopic pregnancies and 53.61±19.15 IU/L for normal/intrauterine pregnancies in studies that used the same unit for reporting this biomarker (2, 4, 6, 7, 9-13, 15-18, 22-24, 27). 

A few studies reported the area under the receiver operating characteristic (ROC) curve of CPK in this regard; Birkhahn et al. (21): 0.56, Ghahiri et al. (25): 0.692, Soundravally et al. (4): 0.851, and Shafi et al. (7): 0.864. According to these reported results, mean area under the ROC curve of CPK was 0.72 in diagnosing ectopic pregnancies.

**Figure:**
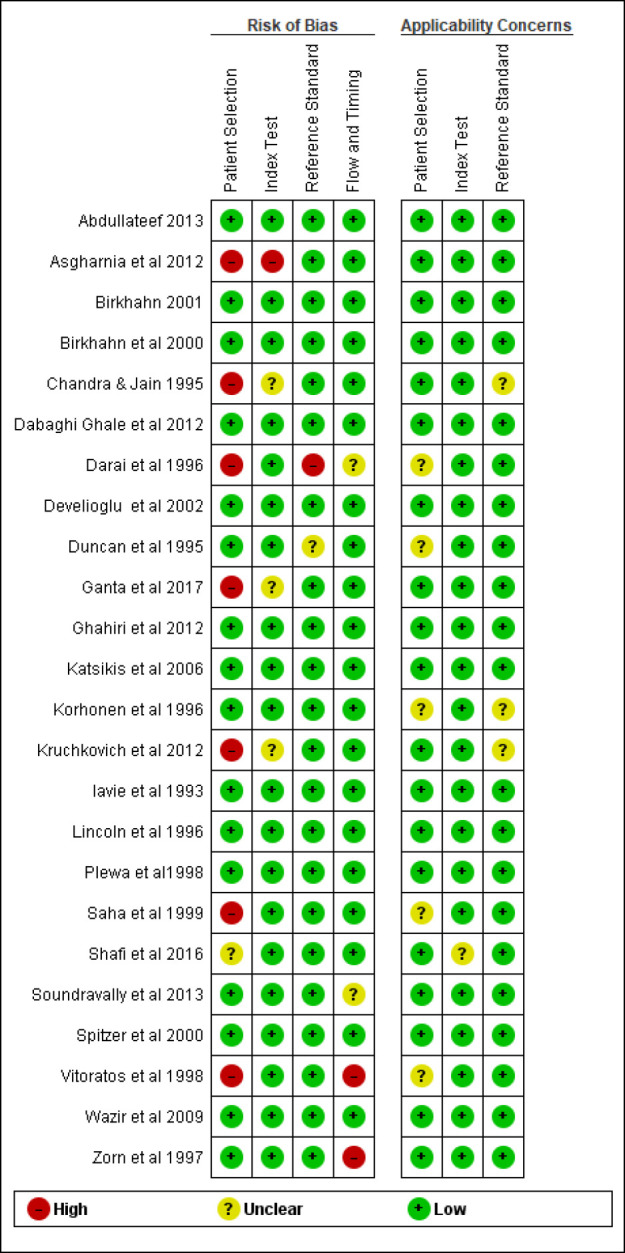


**Table 1:** Characteristics of studies included in the review

NO	1st author, Year of publication	Research information							
**1**	**Lavie et al. (9)1993**	**Mean age of participants: **	Not reported						
		**Mean gestational age: **	Not reported						
		**Total sample size:**	51						
		**Groups of study: **	3 groups of women: Tubal pregnancy (n=17) Missed abortion (n=17) Normal pregnancy (n=17)						
		**Method of measuring CPK: **	Reflectance spectrophotometry of chromophore production.						
		**Time of sampling: **	On admission						
		**Reference test to confirm ectopic pregnancy:**	Physical examination, ultrasonographic examination, and routine blood tests						
		**Mean level (±SD) of CPK in Intact Ectopic pregnancies(IU/l): **	78.125 ± 6.369						
		**Mean level(±SD) of CPK in intra uterine pregnancies(IU/l): **	31.125 ±2.213						
		**Mean level(±SD) of CPK in missed abortions(IU/L): **	26 .25 ± 6.149						
		**p-value: **	p < 0.0001 (**Sig)**						
		**Cut-point **	45** IU/L**						
			**Sensitivity:**					100%	
			**Specificity:**					100%	
**2**	**Duncan et al. (10) 1995**	**Mean age of participants: **	Not reported						
		**Mean gestational age:**	Not reported						
		**Total sample size: **	120						
		**Groups of study: **	4 groups of patient: EP (n=21), complete miscarriage (n=47), incomplete miscarriage (n=32), ongoing pregnancies (n=20).						
		**Method of measuring CPK: **	Hitachi discrete analyzer						
		**Time of sampling: **	on admission						
		**Reference test to confirm ectopic pregnancy: **	Initial clinical assessment and plasma hCG concentration, laparoscopy or laparotomy, histological assessment.						
		**Mean level of CPK in Intact Ectopic pregnancy(IU/l): **	53.4						
		**Mean level of CPK in complete miscarriage:**	37.7						
		**Mean level of CPK in intra uterine pregnancy(IU/l): **	42						
		**P –value:**	P < 0.001 (**Sig)**						
		**Cut-point:**	45 IU/L						
			**Sensitivity :**		0.57				
			**Specificity :**		0.67				
**3**	**Chandra & Jain (11) 1995**	**Mean age of participants:**	Not reported						
		** Mean gestational age: **	6-8 weeks						
		**Total sample size: **	90						
		**Groups of study:**	5 groups of patients; Normal pregnancy (n=20), Missed abortion(n=20), Tubal pregnancy (n=20), Acute appendicitis (n=10) Pelvic inflammatory disease (n=20).						
		**Method of measuring CPK: **	Beckman CX- 5 Synchron (Brea, CA) fully automated clinical chemistry analyzer.						
		**Time of sampling: **	before any surgical intervention						
		**Reference test to confirm ectopic pregnancy: **	Physical examinations were carried out along with routine blood tests and ultrasonographic examination.						
		**Mean level (±SD) of CPK in Intact Ectopic pregnancy(IU/l):**	126±51.78						
		**Mean level(±SD) of CPK in missed abortion:**	41±9.7						
		**Mean level(±SD) of CPK in pelvic inflammatory disease:**	46±9.7						
		**Mean level(±SD) of CPK in acute appendicitis:**	52±14.38						
		**Mean level(±SD) of CPK in normal pregnancy:**	42±9.34						
		**P-value:**	P < 0.0001, (**Sig).**						
**4**	**Darai et al. (12) 1996**	**Mean age of participants: **	Not reported						
		**Mean gestational age in EP: **	47.6 days						
		**Mean gestational age in ongoing pregnancy: **	49.3 days						
		**Mean gestational age in ** **missed abortion: **	48.7 days						
		**No statistically significant difference in GA was found between the 3 groups.**							
		**Total sample size:**	90						
		**Groups of study:**	3 groups of women: Tubal pregnancy (n=30) Ongoing pregnancy(n=30), spontaneous Miscarriage(n=30)						
		**Method of measuring CPK: **	multiparametric analyser (Hitachi 737) with CK N- acetyl cysteine reagents (Boehringer Mannheim UK Ltd, Leves, UK)						
		**Time of sampling: **	before any surgical intervention						
		**Reference test to confirm ectopic pregnancy: **	progesterone, beta-hCG, pelvic sonography						
		**Mean level (±SD) of CPK in Intact Ectopic pregnancies(IU/l): **	81.4±66.2 IU/L						
		**Mean level(±SD) in missed abortions (IU/L): **	84.8±49.3						
		**Mean level(±SD) of CPK in ongoing pregnancies(IU/l): **	81.5±40.3						
		**p-value: **	Not reported						
			There was no statistically significant difference in these 3 groups. (NS)						
			There was no significant difference in CK level between patients with rupture of tubal wall and patients without rupture. (NS)						
			There was not any difference in CKMB level between 3 groups of patients. (NS)						
**5**	**Lincoln et al. (13) 1996**	**Mean age of participants: **	Not reported						
		**Mean gestational age:**	First-trimester						
		**Total sample size: **	51						
		**Groups of study: **	3 groups of patients: spontaneous abortion (n=16) EP (n=18) ongoing pregnancy (n=17)						
		**Method of measuring CPK: **	Ektachem 700 discrete analyzer (Eastman Kodak Co., Rochester, NY).						
		**Time of sampling: **	on admission						
		**Reference test to confirm ectopic pregnancy: **	Quantitative hCG level, transvaginal sonography and surgery.						
		**Mean level (±SD) of CPK in Intact Ectopic pregnancies(IU/l): **	90.6±15.9						
		**Mean level(±SD)of CPK in ongoing pregnancies(IU/l):**	78±13.8						
		** Mean level(±SD)of CPK in abortions(IU/l): **	94.1±13						
		**p-value:**	p>0.7 (NS)						
		**AUC** **:**	0.501 ± 0.007.						
		**p-value:**	P > 0.05, (NS).						
**6**	**Korhonen et al. (14) 1996**	**Mean age of participants:**	Not reported						
		** Mean gestational age in EP group:**	44.9 ± 9.9 days						
		**Mean gestational age in normal pregnancy group;**	37.5 ± 4.0						
		**Total sample size: **	44						
		**Method of measuring CPK: **	Hitachi 911 discrete analyzer using CK N-acetyl cysteine activation reagents at 37°C						
		**Time of sampling: **	On admission or after repeated examinations						
		**Groups of study: **	3 groups of patients: Spontaneous abortion, blighted ovum, or Missed abortion (n=15) Tubal pregnancy (n=15) Normal intrauterine pregnancy (n=14).						
		**Reference test to confirm ectopic pregnancy: **	Transvaginal sonography, laparoscopy and serum hCG						
		**Mean level (±SD) of CPK in Intact Ectopic pregnancies(IU/l):**	Not reported						
		**Mean level (±SD) of CPK in intra uterine pregnancies(IU/l):**	Not reported						
		**Mean level (±SD) of CPK in missed abortions(IU/l): **	Not reported						
		**p-value: **	Not reported						
			No significant differences in CK levels were observed between the groups. (NS)						
		**Cut-point: **	30 IU/L						
			**p-value:**					P < 0.005, (**Sig**.)	
			**Sensitivity:**					Not reported	
			**Specificity:**					Not reported	
**7**	**Zorn et al. (15) ** **1997**	**Mean age of participants: **	Not reported						
		**Mean gestational age: **	Not reported						
		**Total sample size: **	57						
		**Groups of study: **	3 groups of patients: normal pregnancy (n=20), miscarriage (n=23), EP (n=14).						
		**Method of measuring CPK: **	Hitachi 717 analyzer						
		**Time of sampling: **	On admission						
		**Reference test to confirm ectopic pregnancy: **	B-hCG						
		**Mean level (±SD) of CPK in Intact Ectopic pregnancies(IU/l):**	44.2±3.6						
		**Mean level(±SD) of CPK in intra uterine pregnancies(IU/l):**	36.8±5.1						
		**Mean level(±SD) of CPK in intra miscarriages(IU/l):**	51.7±7.2						
		**p-value: **	Not reported						
			There was no significant difference between either normals and miscarriages or miscarriages and ectopics.						
		**Cut-point:**	> 45 IU/L						
			**p-value:**		P < 0.02 (**Sig**)				
			**Sensitivity:**		0.50				
			**Specificity:**		0.76				
			**PPV:**		0.69				
**8**	**Plewa et al. (16) 1998**	**Mean age of participants:**	Not reported						
		**Mean gestational age in participants: **	7.3± 2.3 weeks						
		**Total sample size: **	64						
		**Groups of study: **	3 groups of patients: EP (n=15) Threatened miscarriage (n=28) Normal pregnancy (n=21).						
		**Method of sampling CPK: **	CK was spectrophotometrically assayed with standard reagents by an enzymatic rate method on a Synchron CX System						
		**Time of sampling: **	Initial presentation						
		**Reference test to confirm ectopic pregnancy:**	quantitative beta-hCG Laparoscopy, ultrasonography, dilatation and curettage						
		**Mean level (±SD) of CPK in Intact Ectopic pregnancy(IU/l):**	88.8±33.6						
		** Mean level (±SD) of CPK in intra uterine pregnancy(IU/l):**	56±38.1						
		**Mean level (±SD) of CPK threatened miscarriage (IU/l):**	65.9±59						
		**p-value:**	p=0.02, (**Sig**)						
		**Cut-point.**	≥ 74 IU/L						
		**Sensitivity: **	Not reported						
		**Specificity: **	Not reported						
**9**	**Vitoratos et al. (17) 1998**	**Mean age of participants: **	Not reported						
		**Mean gestational age: **	Not reported						
		**Total sample size: **	66						
		**Groups of study: **	3 groups of patients; EP (n=21) Normal pregnancies (n=20) Abortion (n=15).						
		**Method of measuring CPK: **	Not reported						
		**Time of sampling: **	Not reported						
		**Reference test to confirm ectopic pregnancy: **	beta-hCG, sonography						
		**Mean level (±SD) of CPK in asymptomatic Ectopic pregnancies(U/l):**	58.5 ± 12.42						
		**Mean level (±SD)of CPK in symptomatic Ectopic pregnancies(U/l): **	59 ±10.08						
		**p-value:**	p = 0.45, (NS)						
		**1.Mean level (±SD) of CPK in normal pregnancies (U/l):**	58.5 ± 7.24						
		**2.Mean level (±SD) of CPK in threatened abortion (U/l): **	73 ± 11.43						
		**Cut-point: **	145 IU/l						
		**Sensitivity: **	Not reported						
		**Specificity: **	Not reported						
**10**	**Saha et al. (18) 1999**	**Mean age of participants: **	Not reported						
		**Mean gestational age:**	Not reported						
		**Total sample size: **	40						
		**Groups of study: **	2groups of patients: EP (n=20) Normal pregnancy (n=20).						
		**Method of sampling CPK: **	Kontron-890 spectrophotometer						
		**Time of sampling: **	before any invasive procedure						
		**Reference test to confirm ectopic pregnancy: **	Physical examination, routine blood test, ultrasonographic examination and urine pregnancy test.						
		**Mean level (±SD) of CPK in Intact Ectopic pregnancy(IU/l): **	34.15± 1.17						
		** Mean level (±SD) of CPK in intra uterine pregnancy(IU/l): **	18.72 ± 1.25						
		**p-value:**	p˂ 0.001, (**Sig**)						
		**Cut-point:**	Not-reported						
		**sensitivity: **	Not reported						
		**specificity: **	Not reported						
**11**	**Spitzer et al. (19) 2000**	**Mean age of participants: **	Not reported						
		**Mean gestational age:**	First trimester						
		**Total sample size: **	65						
		**Groups of study: **	3 groups of patients: EP (n=23) Normal pregnancy (n=21) Abnormal pregnancy (n=21).						
		**Method of sampling CPK: **	Hitachi discrete analyzer using creatine ki-nase N-acetyl cysteine activation reagents at 37						
		**Time of sampling: **	On admission						
		**Reference test to confirm ectopic pregnancy: **	Transvaginal ultrasound examination and blood sample for evaluating progesterone, 17-hydroxyprogesterone, CA-125 and androstenedione						
		** Result in patients with 45days of amenorrhea:**							
		**Mean level (±SD) of CPK in Intact Ectopic pregnancies(IU/l):**	88.6/96.3± 37.4						
		**Mean level (±SD) of CPK in normal pregnancies(IU/l):**	54/54.1 ± 170						
		**Mean level (±SD) of CPK in abnormal pregnancies(IU/l): **	77/78.1 ± 46.4						
		**p-value: **	p < 0.002						
		**Result in patients With 2500 MIU/ML B-hCG:**							
		**Mean level (±SD) of CPK in Intact Ectopic pregnancies(IU/l): **	84.5/88.1± 31.8						
		**Mean level (±SD) of CPK in normal pregnancies(IU/l): **	30.6						
		**Mean level (±SD) of CPK in abnormal pregnancies(IU/l): **	61/62.5± 24.7						
		**Cut-point:**	P>0.50 **IU/L**						
			**Sensitivity:**	94%					
			**Specificity:**	31%					
		**Cut-point:**	P>0.70** IU/L**						
			**Sensitivity :**	78%					
			**Specificity:**	81%					
**12**	Birkhahn* et al. (20) **2000** *In this article different unit (mIU/dl) has been used for measuring CPK	**Mean age of participants in EP group: **	31.2±5.8						
		**Mean age of participants in non EP group:**	29.4±6.1						
		**Mean gestational age:**	First trimester						
		**Total sample size:**	42						
		**Groups of study: **	2 groups of patients: EP (n=21) Non-EP (n=21).						
		**Method of measuring CPK: **	Not reported						
		**Time of sampling: **	At the time of presentation						
		**Reference test to confirm ectopic pregnancy:**	Ultrasonography, laparoscopy, or laparotomy.						
		**Mean level (±SD) of CPK in Intact Ectopic pregnancies (mIU/dl):**	118±47						
		**Mean level (±SD) of CPK in nonEps ** ***(mIU/dl)*** **: **	64±45.3						
		**p-value:**	P < .0031, (**Sig**)						
		**Cut-point:**	70 mIU/dL						
			**Sensitivity:**			100%			
			**Specificity:**			61.9%			
			**PPV:**			72.4%			
			**NPV:**			100%			
**13**	Birkhahn* et al. (21) 2001 *In this article different unit (ng/dl) has been used for measuring of CPK	**Mean age of participants in EP group: **	31.9						
		**Mean age of participants in non-EP group** **:**	27.8						
		**Mean gestational age: **	First trimester						
		**Total sample size: **	378						
		**Groups of study: **	2 groups of patients: patients at less than 5 weeks’ gestation and EP(n=61: ruptured=24 or unruptured=37), non-ectopic pregnancy(n=317; spontaneous abortion, threatened, incomplete, missed, and complete, urinary tract infection, corpus luteal cyst, nonspecific abdominal pain, renal cyst, pelvic inflammatory disease, or appendicitis ).						
		**Method of measuring CPK: **	OPERA, Bayer Corporation, Pittsburgh, PA						
		**Time of sampling: **	At the time of presentation						
		**Reference test to confirm ectopic pregnancy: **	Surgical pathology reports or ultrasonographic scans, β-hCG, SMHC and myoglobin test.						
		**Mean level (95% CI) of CPK in Ectopic pregnancies (ng/dl): **	119 (89–149)						
		**Mean level (95% CI) of CPK in unruptured Ectopic pregnancies (ng/dl): **	104 (85–122)						
		**Mean level (95% CI) of CPK in ruptured Ectopic pregnancies (ng/dl): **	139 (66–212)						
		**Mean level (95% CI) of CPK in non-Ectopic pregnancies (ng/dl):**	105 (95–115)						
		**p-value: **	Not reported						
		**The AUC for total creatine kinase: **	0.56 (95% CI0.51 to 0.61)						
		**Cut-point: **	1.1 μg/L						
			**Sensitivity:**						72%
			**Specificity:**						52%
		**The AUC for SMHC: **	0.63						
14	**Develioglu et al. (6) 2002**	**Mean age of participants in isthmic EP group:**	34±3.3						
		**Mean age of participants in ampullary EP group:**	31.5±5.1						
		**Mean gestational age in isthmic EP** **:**	35.8±3.7 days						
		**Mean gestational age in ampullary EP** **:**	37.6±5 days						
		**Total sample size: **	52						
		**Groups of study: **	2 groups of patients: EP (n=32) Intrauterine pregnancies (n=20).						
		**Method of measuring CPK: **	spectrophotometric method on a Technicon DAX Systems automated analyzer						
		**Time of sampling: **	On admission						
		**Reference test: **	beta-hCG, ultrasound, endometrial biopsy, laparoscopy or laparotomy.						
		**Mean level (±SD) of CPK in in isthmic EP(IU/l): **	185.6±58.3						
		**Mean level (±SD) of CPK in in ampullary EP(IU/l): **	112.3±55.5						
		**p-value:**	P=0.011, (**Sig**)						
		**Mean level (±SD) of CPK in in ruptured EP(IU/l): **	152.1±61.2						
		**Mean level (±SD) of CPK in in unruptured EP(IU/l): **	91.6±44.3						
		**Mean CK level in normal pregnancy (IU/l): **	77.4±38.2						
		**p-value: **	P˂0.011, (Sig) Comparing CPK levels in isthmic and ampullary ectopic pregnancies.						
			P˂0.003, (Sig) Comparing CPK levels in ruptured ectopic pregnancy and unruptured						
			P<0.0001, (Sig). Comparing CPK levels in unruptured ectopic pregnancy and normal pregnancy.						
		**Cut-point:**	>120 Iu/L						
			**Sensitivity:**		65%				
			**Specificity:**		87%				
**15**	**Katsikis et al. (22) 2006**	**Mean age of participants in EP group: **	28.1±6.3						
		**Mean age of participants in abortion group:**	26.9±4.9						
		**Mean age of participants in normal pregnancy:**	24.6±2.7						
		**Mean gestational age:**	First trimester						
		**Total sample size: **	80						
		**Groups of study: **	3 groups of patients: EP (n=40) Abortion (n=20) Normal intrauterine pregnancy (n=20).						
		**Method of measuring CPK: **	enzyme-linked immunoassay (ELISA)						
		**Time of sampling:**	at the time of presentation and 24 hours after surgery in the subgroups of women with EP and Intra uterine abortion						
		**Reference test to confirm ectopic pregnancy: **	Clinical assessment and transvaginal ultrasonography, beta-hCG						
		**Mean level (±SD) of CPK in Ectopic pregnancies (U/l): **	33.4±15.4						
		**Mean level (±SD)of CPK in normal pregnancies (U/l):**	24.7±5.7						
		**Mean level (±SD)of CPK in abortions (U/l): **	17.9±3.7						
		**p-value of comparing CPK in EP with both IU abortions:**	p˂0.001, (**Sig**).						
		**p-value of comparing CPK in EP with normal gestations:**	p˂0.01, (**Sig**).						
		**p-value of comparing CPK-MB in normal pregnancy compared with EP or IU abortion:**	p˂0.01, (**Sig**).						
		**Cut-point:**	>26.5 U/L						
			**Sensitivity:**			80%			
			**Specificity:**			87.5%			
			**PPV:**			86.5%			
			**NPV:**			81.4%			
		**Cut-point:**	˂ 14.4 U/L						
			**Sensitivity:**			82.5%			
			**Specificity:**			95%			
			**PPV:**			94.3%			
			**NPV:**			84%			
16	**Wazir et al. (23) 2009**	**Mean age of participants:**	Not reported						
		**Mean gestational age: **	First trimester						
		**Total sample size: **	100						
		**Groups of study: **	2 groups of patients: Tubal pregnancy (n=50) Intra uterine pregnancy (n=50).						
		**Method of measuring CPK: **	Not reported						
		**Time of sampling: **	On admission						
		**Reference test to confirm ectopic pregnancy: **	physical examination, routine investigations and ultrasonography of abdomen, transvaginal ultrasound, laparoscopy and/ or laparotomy						
		**Mean level (±SD) of CPK in Ectopic pregnancies(IU/l):**	103±50						
		**Mean level (±SD) of CPK in normal pregnancies(IU/l):**	52.4 ±10.9						
		**p-value:**	P=0.000, (**Sig**).						
		**Mean level (±SD) of CPK in ruptured Ectopic pregnancies(IU/l):**	119.8±70.5						
		**Mean level (±SD) of CPK in unruptured Ectopic pregnancies(IU/l):**	88.6±19.6						
		**P-value:**	P=0.002, (**Sig**).						
		**Cut-point:**	70 IU/L						
			**Sensitivity:**					95%	
			**Specificity:**					98%	
			**PPV:**					99%	
			**NPV:**					90.7%	
**17**	**Elmizadeh et al. (24)** **2012**	**Mean age of participants in Ep group:**	27.4±6.1						
		**Mean age of participants in intrauterine pregnancy:**	26.4±6.5						
		**Mean gestational age in EP group:**	7.9±1.8 weeks						
		**Mean gestational age in intrauterine pregnancy:**	8.5±1.7						
		**Total sample size:**	55						
		**Groups of study:**	2 groups of patients: EP (26), Normal Intrauterine pregnancy (29).						
		**Method of measuring CPK: **	Kinetic UV-Method						
		**Time of sampling: **	Before any invasive intervention						
		**Reference test to confirm ectopic pregnancy: **	Not reported						
		**Mean level (±SD) of CPK in tubal Ectopic pregnancies(IU/l): **	156±93.6						
		**Mean level (±SD)of CPK in intra uterine pregnancies(IU/l):**	58.63±31.5						
		**p-value:**	p˂0.0001, (**Sig)**.						
		**Mean level (±SD) of CPK in Intact Ectopic pregnancy(IU/l): **	104.3±21.4						
		**Mean level (±SD) of CPK in ruptured Ectopic pregnancy(IU/l): **	220.8±100.5						
		**P-value:**	P=0.002, (**Sig**).						
		**sensitivity:**	92%						
		**specificity:**	86%						
		**PPV:**	85.7%						
		**NPV:**	92.5%						
**18**	**Asgharnia et al. (2) 2012**	**Mean age of participants: **	16-40						
		**Mean gestational age: **	First-trimester						
		**Total sample size: **	111						
		**Groups of study: **	3 groups of study; Tubal ectopic pregnancy (n=37) Threatened abortion (n=37) Normal intra-uterine pregnancy (n=37).						
		**Method for measuring CPK: **	Photometric pars-azmun kit at 37oC						
		**Time of sampling:**	Before any invasive procedure						
		**Reference test to confirm ectopic pregnancy: **	Sonography and β-hCG level						
		**Mean level (±SD) of CPK in Intact Ectopic pregnancies (IU/l):**	96.27±63.9						
		**Mean level (±SD) of CPK in normal pregnancies (IU/l):**	48.94±19.2						
		**Mean level (±SD) of CPK in threatened abortions (IU/l):**	55.37±14.1						
		**p-value:**	p<0.0001, (**Sig**).						
		**Mean level (±SD) of CPK-MB in Ectopic pregnancies (IU/l): **	15.62±5.2						
		**Mean level (±SD) of CPK-MB in threatened abortions (IU/l) : **	17.32±6.9						
		**Mean level (±SD) of CPK-MB in normal pregnancies (IU/l):**	15.1±4.7						
		**P-value: **	P=0.219, (NS).						
		**Sensitivity: **	Not reported						
		**Specificity: **	Not reported						
**19**	**Kruchkovich et al. (8) 2012**	**Mean age of participants:**	29.2 years						
		**Mean gestational age:**	5.6 weeks						
		**Total sample size: **	79						
		**Groups of study: **	2 groups of patients; EP (n=51) Normal pregnancy (n=28).						
		**Method for measuring CPK: **	Kinetic UV test						
		**Time of sampling: **	On admission						
		**Reference test to confirm ectopic pregnancy**	Vaginal ultrasound and serial beta hCG testing.						
		**Mean level (±SD) of CPK in Intact Ectopic pregnancies(U/l):**	80.90±62.13						
		**Mean level(±SD) of CPK in intra uterine pregnancies (U/l): **	74.9±51.6						
		**P-value:**	P=0.66, (NS).						
		**Mean level (±SD) of CPK-MB in Intact Ectopic pregnancies(U/l): **	11.1 ± 8.2						
		**Mean level (±SD) of CPK-MB in intra uterine pregnancies (U/l): **	74.9±51.6						
		**P-value:**	0.39						
		**Sensitivity: **	Not reported						
		**Specificity: **	Not reported						
**20**	**Ghahiri et al. (25) 2012**	**Mean age of participants of EP group: **	28.62 ± 4.97						
		**Mean age of participants of non-EP group:**	27.58 ± 4.53						
		**Mean gestational age: **	First trimester						
		**Total sample size: **	106						
		**Groups of study: **	2 groups of patients; EP (n=53) Non-EP (n=53).						
		**Method of measuring CPK: **	Not reported						
		**Time of sampling: **	On admission						
		**Reference test to confirm ectopic pregnancy: **	Transvaginal sonography, beta-hCG						
		**Mean level (±SD) of CPK in Intact Ectopic pregnancies(IU/l): **	Not reported						
		**Mean level (±SD) of CPK in non-Ectopic pregnancies(IU/l): there**	Not reported						
		**p-value of CPK level and the type of pregnancy: **	P=0.0001, (**Sig**).						
		**p-value of CPK-MB level and the type of pregnancy:**	P=0.003, (**Sig**).						
		**Mean gestational age: **	First trimester						
		**Total sample size: **	106						
		**Groups of study: **	2 groups of patients; EP (n=53) Non-EP (n=53).						
		**Method of measuring CPK: **	Not reported						
		**Time of sampling: **	On admission						
		**Reference test to confirm ectopic pregnancy: **	Transvaginal sonography, beta-hCG						
		**Mean level (±SD) of CPK in Intact Ectopic pregnancies(IU/l): **	Not reported						
		**Mean level (±SD) of CPK in non-Ectopic pregnancies(IU/l): there**	Not reported						
		**p-value of CPK level and the type of pregnancy: **	P=0.0001, (**Sig**).						
		**Cut-point:**	61 IU/L						
			**Sensitivity:**						
			**Specificity:**						
			**PPV:**						
			**NPV:**						
			**PLR** **:**						
			**NLR** **:**						
			**p-value:**						
		**AUC:**	0.692						
		**Cut-point for CPK-MB:**	15.6 IU/L						
			**Sensitivity:**					71.7%	
			**Specificity:**					56.6%	
			**PPV:**					62.29%	
			**NPV:**					66.7%	
			**PLR :**					1.65	
			**NLR :**					0.5	
		**AUC:**	0.647						
**21**	**Soundravally et al. (4) ** **2013**	**Mean age of participants in abortion group:**	24.2±4						
		**Mean gestational age in normal pregnancy: **	5.4±2.1 weeks						
		**Mean gestational age in abortion: **	5.2±1.2 weeks						
		**Mean gestational age in EP: **	4.6±1.5 weeks						
		**Total sample size: **	63						
		**Groups of study: **	2 groups of patients; Ruptured EP (n=32) Intrauterine abortion and normal pregnancies (n=31).						
		**Method of measuring CPK: **	Immuno-inhibition method, in an auto-mated analyzer						
		**Time of sampling: **	At the time of presentation prior to surgical intervention						
		**Reference test to confirm ectopic pregnancy: **	Clinical assessment, beta-hCG and transvaginal ultrasonography.						
		**Mean level (±SD) of CPK in Intact Ectopic pregnancies(IU/l):**	305±357						
		**Mean level (±SD)of CPK in normal pregnancies(IU/l): **	93±35						
		**Mean level (±SD) of CPK in abortions (IU/l): **	87±71						
		***p-value:***	p< 0.05, (**Sig**).						
		**Mean level (±SD) of CPK-MB% in Intact Ectopic pregnancies: **	6±8						
		**Mean level (±SD)of CPK-MB% in normal pregnancies: **	14±6						
		**Mean level (±SD) of CPK-MB% in abortions: **	19±15						
		***p-value: ***	p< 0.05, (**Sig**).						
		**Cut-point for CPK:**	147 IU/L						
			**Sensitivity:**					72%	
			**Specificity:**					89%	
		**Cut-point for CPK-MM and CPK-MB% respectively:**	132 IU/L, 10						
		**AUC:**	0.882						
			**Sensitivity:**					72%	
			**Specificity:**					93%	
		**AUC:**	0.851						
			**Sensitivity:**					88%	
			**Specificity:**					68%	
**22**	**Abdullateef (26) 2013**	**Mean age of participants in EP group: **	28.97±0.957						
		**Mean age of participants in abortion group: **	32.05±1.95						
		**Mean age of participants in normal pregnancy:**	25.8±1.139						
		**Mean gestational age:**	Not reported						
		**Total sample size: **	81						
		**Groups of study: **	3 groups of patients; EP (n=40) Intrauterine abortion (n=17) , Normal pregnancy (n=24).						
		**Method of measuring CPK: **	Spectrophotometric analysis						
		**Time of sampling: **	On admission						
		**Reference test to confirm ectopic pregnancy:**	Clinical assessment and transvaginal ultrasonography.						
		**Mean level (±SD) of CPK in Intact Ectopic pregnancies(IU/l): **	Not reported						
		**Mean level (±SD) of CPK in intra uterine pregnancies(IU/l): **	Not reported						
		**Mean level (±SD) of CPK in abortions(IU/l): **	Not reported						
		**AUC:**	0.903 (95%CI: 0.831–0.975)						
			p-value:					p< 0.001, (**Sig**).	
		**cut-point for CPK:**	29.43 IU/L						
			**sensitivity:**					92%	
			**specificity:**					100%	
			**PPV:**					100%	
			**NPV:**					96%	
		**Cut-point for CPK-MB:**	4.55 IU/L						
			**sensitivity:**					81.64%	
			**specificity:**					84.3%	
			**PPV:**					71.4%	
			**NPV:**					71.4%	
**23**	**Shafi et al. (7) 2016**	**Mean age of participants: **	Not reported						
		**Mean gestational age: **	Not reported						
		**Total sample size: **	175						
		**Groups of study: **	2 groups of patients; EP (n=100) Normal pregnancy (n=75).						
		**Method of measuring CPK: **	UV kinetic method-NAC						
		**Time of sampling: **	Before any invasive procedure.						
		**Reference test to confirm ectopic pregnancy: **	routine investigations and ultrasonography						
		**Mean level (±SD) of CPK in Intact Ectopic pregnancies(IU/l):**	97.64±33.08						
		**Mean level (±SD) of CPK in normal pregnancies(IU/l):**	53.20±9.75						
		**Mean level (±SD) of CPK in ruptured tubal pregnancies(IU/l): **	111.71±41.56						
		**Mean level (±SD) of CPK in unruptured tubal pregnancies(IU/l): **	84.12±11.36						
		**p-value:**	p˂0.001, (**Sig**).						
		**AUC:**	86.4%						
			**Sensitivity:**				Not reported		
			**Specificity:**				Not reported		
**24**	**Ganta et al. (27) 2017**	**Mean age of participants: **	20-40						
		**Mean gestational age: **	Not reported						
		**Total sample size: **	120						
		**Groups of study: **	3 groups of patients; EP (n=40) Abortion (40) Normal pregnancy (n=40).						
		**Method of measuring CPK: **	NAC activated with Beckman Coulter AU480.						
		**Time of sampling: **	Before any invasive procedure						
		**Reference test to confirm ectopic pregnancy: **	beta-hCG, sonography, Laparoscopy , Laparotomy						
		**Mean level (±SD) of CPK in Intact Ectopic pregnancies(IU/l): **	91.55±30.43						
		**Mean level (±SD) of CPK in ruptured Ectopic pregnancies(IU/l): **	97.26±25.97						
		**Mean level (±SD) of CPK in unruptured Ectopic pregnancies(IU/l): **	63.82±34.9						
		**Mean level (±SD) of CPK in normal pregnancies(IU/l):**	36.92±6.44						
		**Mean level (±SD) of CPK in abortions(IU/l): **	43.95±11.96						
		**p-value of between 3 groups:**	p<0.0001, (**Sig**).						
		**p-value of ruptured and unruptured EP:**	P=0.015, (**Sig**).						
		**sensitivity: **	Not reported						
		**specificity: **	Not reported						

SD: standard deviation; CK/CPK: creatine phosphokinase; EP: ectopic pregnancy; Sig: significant; NS: not significant; AUC: area under the receiver operating characteristic (ROC) curve; PPV: positive predictive value; NPV: negative predictive value; GA: gestational age; SMHC: smooth muscle heavy-chain myosin; PLR: positive likelihood ratio; NLR: negative likelihood ratio; NAC: N-acetyl-cystein.

Studies reported different cut-points for level of CPK in diagnosis of ectopic pregnancy, ranging from 26.5 to more than 145 IU/ with sensitivity and specificity ranging from 65% to 95% and 64.5% to 100%, respectively (2,4, 6,11, 12, 16-19, 24-28). For example, Lavie et al. (9), reported CPK level of 45 IU/L as a cut-point for diagnosing EP with 100% sensitivity and 100% specificity, whereas at the same cut-point, Duncan et al. (10) and Zorn et al. (15) reported sensitivity of 57% and 50%, and specificity of 67% and 76%, respectively. Some studies, only reported the cut-point without calculating sensitivity and specificity; for example Korhonen et al. (14), Plewa et al. (16), and Vitoratos (17), claimed that CPK=30 IU/L, CPK ≥ 74 IU/L, CPK=145 IU/L are suitable for diagnosing ectopic pregnancy, respectively.

## Discussion

The results highlighted the potential benefits of CPK as a marker for early diagnosis of EP. Studies show that the reported prevalence of EP is increasing in different countries in recent decades due to improved initial diagnosis and increased risk factors for EP, especially the use of assisted reproductive technology. Timely diagnosis of EP saves the mother and ensures her health. Therefore, we conducted a systematic review of existing papers to investigate and introduce an applied method for early diagnosis of EP to prevent its complications and consequences. 

CPK is an intracellular enzyme in muscle cells and its plasma level increases in cell lysis. Lysis of trophoblast cells leads to an increase in plasma CPK levels. Therefore, the level of this enzyme can be used for evaluation of tubal ectopic pregnancy because increased CPK can be associated with trophoblastic invasion and trophoblast mass (6). 

Currently, transvaginal ultrasound and serial measurement of serum beta-hCG levels are the most common diagnostic methods for ectopic pregnancy (7). If the ultrasound shows the presence of pregnancy tissue in adnexa without evidence of intrauterine pregnancy, the presence of ectopic pregnancy should be suspected (28), If the yolk sac or embryo are detected in the EP tissue, the diagnosis of EP is confirmed. Serum beta-hCG also plays an important role in diagnosis of EP along with ultrasound (29).

Despite the widespread use of transvaginal ultrasound and beta-hCG, it is believed that 40% to 50% of EP cases remain unidentified in the early stages. Despite the advances in ultrasound, according to recent reports, 48-82% of all patients with abdominal pain or vaginal bleeding in the first trimester of pregnancy have an uncertain ultrasonography with serum beta-hCG level less than 1000 IU/dl. Particularly, this subgroup of patients are not properly evaluated and may benefit most from other serum markers that allow rapid diagnosis (7).

According to the present review, researchers around the world are interested in using CPK as a diagnostic marker for early detection of EP. Asgharnia et al. reported that determining the total CPK level could increase its diagnostic value in diagnosis of tubal ectopic pregnancy; however, the need for larger scale studies was felt to appropriately determine the cut-off point of this marker. They did not find any significant differences between study groups regarding CPK-MB levels (2).

Six of the papers reviewed in this study did not confirm the significance of the differences in CPK levels between those with intrauterine and ectopic pregnancies. According to the studies summarized in Table 1, all of these articles, except for one published in 2012 (8), were published in the years before 2000 (12-17). Kruchkovich et al. reported that no significant difference in CPK and CPK-MB levels had a diagnostic value for EP. They concluded that the inaccuracies observed regarding CPK in that study could be due to the participation of subjects with less than 7 weeks of gestational age because CPK level might also be related to the gestational age (8). Plewa et al. reported that although there are higher levels of CPK in patients with ectopic pregnancy compared to those with abortion or normal pregnancy, due to a significant overlap in CPK levels, the use of this serum marker for EP diagnosis is unreliable (16). A drawback of that study was its small sample size, especially in the EP group, which can reduce the confidence in the confirmation of its findings.

**The strengths and limitations of this study**

- This study examined various aspects of CPK and its evidence in early diagnosis of EP.
- Systematic reviews provide the highest level of evidence for decision-making. To the best of our knowledge, there was no systematic review for the current issue.
- Two independent researchers conducted screening and extraction of studies.

## Conclusions

According to this review, serum CPK level can be used as a diagnostic marker for ectopic pregnancies and it seems that mean level of CPK is 95.02±51.09 IU/L for ectopic pregnancies and 53.61±19.15 IU/L for normal/intrauterine pregnancies. 
